# Integrated morphological, proteomic and metabolomic analyses reveal response mechanisms of microalgae under uranium exposure

**DOI:** 10.3389/fmicb.2025.1679056

**Published:** 2025-10-20

**Authors:** Mengwei Han, Xu Yang, Bin Dong, Jinlong Lai, Hailing Xi, Sanping Zhao

**Affiliations:** ^1^State Key Laboratory of Chemistry for NBC Hazards Protection, Beijing, China; ^2^School of Life Sciences, Southwest University of Science and Technology, Mianyang, China; ^3^Engineering Research Center of Biomass Materials, Ministry of Education, Southwest University of Science and Technology, Mianyang, China

**Keywords:** microalgae, uranium, antioxidative enzyme, proteomic and metabolomic analysis, bioremediation

## Abstract

**Introduction:**

The release of uranium (U)-containing wastewater poses a significant threat to aquatic ecosystems. However, the response mechanisms of microalgae to U stress remain poorly understood.

**Methods:**

This study employed an integrated approach, combining morphological, physiological, proteomic, and metabolomic analyses, to investigate the tolerance and accumulation mechanisms of the microalga *Ulothrix* sp. under a gradient of U exposure (40 to 400 μmol/L).

**Results:**

*Ulothrix* sp. exhibited a dose-dependent U accumulation, reaching up to 2,100 mg/kg dry weight, which competitively inhibited the uptake of phosphorus and zinc. Physiologically, medium and high U concentrations reduced chlorophyll *a* content by 49–65% and significantly impaired photosystem II efficiency (Fv/Fm), while increasing energy dissipation (DIo/RC) by 1.82–2.01 times. Antioxidant defense responses were activated, with significant upregulation of superoxide dismutase (SOD) and catalase (CAT) activities (*p* < 0.05), a 2.2-fold increase in oxidized glutathione (GSSG), and inhibition of peroxidase (POD) activity. Proteomic analysis revealed that differentially expressed proteins (DEPs) were predominantly enriched in pathways related to carbohydrate metabolism and transport. Concurrently, metabolomic profiling indicated a specific activation of the glycine-serine-threonine pathway and a significant enrichment in glycerophospholipid metabolism (ko00564).

**Discussion:**

Our findings demonstrate that *Ulothrix* sp. mitigates U-induced stress and maintains cellular homeostasis through a multi-level defense network. This network encompasses the activation of antioxidant enzymes, remodeling of key tricarboxylic acid (TCA) cycle metabolites, and strategic regulation of the glycine-serine-threonine metabolic pathway. This study provides crucial insights into the molecular and physiological basis of U tolerance in microalgae.

## 1 Introduction

With the rapid development of the nuclear industry, uranium (U) pollution has become a significant global environmental concern (Ma et al., 2020). Nuclear energy production processes, such as mining, uranium hydrometallurgy, and nuclear fuel manufacturing, generate large amounts of U-containing wastewater that is discharged into the environment (Zhu et al., 2022). Increasing U concentrations in water pose a threat to human health; the combined chemical and radiological toxicity of U present serious ecological risks (Parmar and Patel, 2025), making its environmental behaviors and ecological risks a significant focus. Since its chemical toxicity is significantly higher than that of its radioactive isotopes, chemical toxicity is particularly substantial in biological systems (Chen et al., 2024).

It is well-established that microorganisms play a vital role in the remediation of water contaminated (Chen et al., 2025; Zhang et al., 2025a). Studies have shown that live microalgae cells (e.g., *Chlorella, Spirulina*) can effectively adsorb uranium (U) through mechanisms such as cell wall complexation ion exchange, and intracellular accumulation (Vogel et al., 2010; Embaby et al., 2022). For instance, *Ankistrodesmus* sp. achieved a maximum removal rate of 98% at a concentration of 80 mg/L U (Cheng et al., 2023). Moreover, microalgae counteract stress by activating antioxidant enzyme systems (such as SOD, CAT, and POD) and reprogramming their metabolism to maintain cellular homeostasis (Tan et al., 2024). These inherent characteristics render microalgae promising biological agents for the bioremediation of uranium-contaminated aquatic systems (John et al., 2022). Understanding of U toxicity and the molecular mechanisms underlying U stress has increased significantly in recent years (Sook et al., 2024). For example, U enters cells via calcium ion channels (Sarthou et al., 2022) and endocytosis. Carboxyl, amino, and organic/inorganic phosphate groups on the microalgal cell wall participate in U complexation (Plöhn et al., 2021). A synergistic approach to uranium immobilization through biomineralization, biosorption, and bioreduction (Liang et al., 2025). U stress causes significant damage to cell division (Lavoie et al., 2014; Feng et al., 2023) and inhibits photosynthesis (Herlory et al., 2013). Microalgae utilize the regulation of oxidase activity to cope with heavy metal stress (Huang et al., 2023; Qiu et al., 2022). Their stress metabolic adaptations involve the accumulation of lipids and carotenoids, and they can experience extensive cell lysis followed by natural regeneration after prolonged U exposure and U biomineralization (Acharya et al., 2017). However, existing research predominantly focuses on single physiological or biochemical aspects (e.g., antioxidant enzyme activity or adsorption capacity) or single-omics levels. A lack of systematic understanding remains regarding the response mechanisms of multi-level regulatory networks (e.g., protein-metabolite interactions) under U stress. Additionally, most studies focus on cyanobacteria or algae (e.g., Anabaena, Chlorella), with limited systematic research on filamentous microalgae in response to U stress. The filamentous structure of *Ulothrix* sp. provides a significantly higher surface area than that of unicellular microalgae, such as Chlorella. This morphological trait enables highly efficient biosorption of uranium onto cell surfaces. In addition, the filamentous morphology facilitates easier harvesting and recovery. Furthermore, *Ulothrix* sp. exhibits strong resilience to environmental stressors, supporting its potential application under real remediation conditions (Zhang and Luo, 2022).

Therefore, this study selected the filamentous microalgae *Ulothrix* sp. as the subject of research. Exposure experiments were conducted with low (40 μmol/L), medium (200 μmol/L), and high (400 μmol/L) U concentrations. Multidimensional data integrating growth physiology and biochemical indicators, elemental analysis, proteomics, and metabolomics were analyzed to investigate the characteristics of intracellular uranium (U) enrichment in *Ulothrix* sp.

## 2 Materials and methods

### 2.1 Experimental design and microalgae growth monitoring

The freshwater microalgae *Ulothrix* sp. (obtained from the Freshwater Algae Culture Collection at the Institute of Hydrobiology, Chinese Academy of Sciences) were selected as the experimental material. The microalgae were cultured in sterile BG-11 medium under controlled conditions at 25 ± 2 °C, with a light intensity of 4,000 lux and a 12 h:12 h light/dark cycle for 7 days.

Uranium exposure was applied in the form of uranyl nitrate [UO₂(NO₃)₂], with concentrations set according to previous literature (Cardenas et al., 2008) as follows: control (U0, 0 mmol/L), low concentration (U1, 40 μmol/L), medium concentration (U2, 200 μmol/L), and high concentration (U3, 400 μmol/L). Microalgae culture (10 mL) in the logarithmic growth phase (initial OD₆₈₀ ≈ 0.8) was into each experimental group. All treatments included three independent biological replicates to ensure statistical reliability (Lai et al., 2024; Zhao et al., 2024).

Two milliliters of microalgae culture from each treatment group were sampled daily at a fixed time. The optical density at 680 nm (OD₆₈₀) was measured using a Multiskan FC microplate reader (Thermo Fisher Scientific, United States) with sterile BG-11 medium as the blank control. Microalgae cell density was calculated according to the standard curve regression equation: y (×10^5^ cells/mL) = 34.1x + 0.7 (*R*^2^ = 0.9917), where x is the OD₆₈₀ value and y is the cell density (×10^5^ cells/mL) (Song et al., 2017).

### 2.2 Chlorophyll and carotenoid extraction and quantification

A 5 mL aliquot of microalgae culture from each treatment group was mixed with 5 mL of 95% (v/v) ethanol and an appropriate amount of quartz sand. The mixture was thoroughly ground, followed by extraction in the dark at 4 °C for 24 h. The extract was then adjusted to a final volume of 10 mL, centrifuged at 3,000 rpm (2,000 × g) for 5 min, and the supernatant was collected. Absorbance was measured at 665 nm (A₆₆₅), 720 nm (A₇₂₀), and 470 nm (A₄₇₀) using a Cary 60 UV–Vis spectrophotometer (Agilent, United States). Chlorophyll a (Chl a) and carotenoid concentrations were calculated using the following equations (Castle et al., 2011; Sujetoviene and Galinyte, 2016):


Chla(μg/mL)=12.9447×(A665−A720)

Carotenoids(μg/mL)=(4.1×A470)−(0.0435×Chla)

### 2.3 Analysis of chlorophyll fluorescence parameters

Rapid chlorophyll fluorescence induction kinetics (OJIP) were analyzed using a handheld chlorophyll fluorometer (FluorPen FP 110, Photon Systems Instruments, Czech Republic). Before measurement, 5 mL of microalgae culture was dark-adapted for 30 min to ensure steady-state photosynthetic conditions. Key fluorescence parameters were recorded, including: maximum photon yield (Fv/Fm), absorbed photon flux per unit photoreaction center (ABS/RC), unit Initial electron transfer photon flux of photoreaction center (ETo/RC), initial capture photon flux per unit photoreaction center (TRo/RC), energy dissipation per unit reaction center (DIo/RC), light absorption performance index (Pi_Abs) (Yang et al., 2019).

### 2.4 Micro-morphology and FT-IR analysis of *Ulothrix* sp.

Microalgae cells exposed for 7 days were collected and processed for micro-morphological analysis. Samples underwent fixation, dehydration, critical point drying, and gold sputter coating to preserve surface structures and enhance conductivity. Micro-morphological observations of *Ulothrix* cells were conducted using a Regulus 8100 field emission scanning electron microscope (FE-SEM, HITACHI, Japan). Simultaneously, semi-quantitative elemental analysis was performed using an attached energy-dispersive X-ray spectroscopy (EDS) system to identify the types of elements and determine their relative abundances in specific micro-regions of the cell surface.

For compositional analysis, freeze-dried algal samples were mixed with potassium bromide (KBr) at a mass ratio of approximately 1:100, thoroughly ground, and pressed into pellets. FT-IR spectra were acquired using an ALPHA II Fourier transform infrared spectrometer (Bruker, Germany) to characterize the structural features of the major functional groups in the microalgae cells.

### 2.5 Analysis of oxidative stress-related enzyme activities in microalgae

After 7 days of uranium (U) exposure, microalgae cultures from each treatment group (U0, U2, U3) were centrifuged at 4 °C and 800 × g for 5 min to harvest the microalgae cells. The collected cells were washed twice with ice-cold phosphate-buffered saline (PBS, pH 7.4) to remove residual culture medium. Microalgae cell lysates were prepared, and the activities of catalase (CAT), superoxide dismutase (SOD), and peroxidase (POD), as well as the content of oxidized glutathione (GSSG), were measured using commercial assay kits (Solarbio Science and Technology Co., Ltd., Beijing, China) following the protocols. All biochemical parameters were normalized to cell number (expressed as enzyme activity units per 10^6^ cells or GSSG content in nmol per 10^6^ cells) before statistical analysis to account for potential variations in cell density.

### 2.6 Elemental content analysis in microalgae biomass

Following 7 days of uranium exposure, microalgae cells were harvested by vacuum filtration and subsequently rinsed three times with sterile phosphate-buffered saline (PBS, pH 7.4) to remove residual culture medium. The collected biomass was subjected to freeze-drying and ground into a homogeneous fine powder. Approximately 0.1 g of freeze-dried microalgae powder was precisely weighed into a digestion vessel. A mixture of 5 mL concentrated nitric acid (HNO₃, GR) and 2 mL hydrogen peroxide (H₂O₂, 30%, GR) was added, followed by closed-vessel digestion in a graphite furnace digestion system at 120 °C until the solution achieved complete clarity and transparency. After cooling to ambient temperature, the digested samples were diluted to a fixed volume with ultrapure water.

Elemental analysis was performed using inductively coupled plasma mass spectrometry (ICP-MS, NexIon 1000G, PerkinElmer, United States) to quantify uranium (U) and mineral elements, including potassium (K), phosphorus (P), sulfur (S), iron (Fe), nickel (Ni), zinc (Zn), copper (Cu), and molybdenum (Mo). Each treatment group was analyzed with three independent biological replicates to ensure statistical reliability.

### 2.7 Quantitative proteomics analysis

The proteomic workflow comprised protein extraction, quantification, in-solution digestion, peptide desalting, and liquid chromatography–tandem mass spectrometry (LC–MS/MS) analysis using a timsTOF HT mass spectrometer (Bruker). Data acquisition was performed in data-independent acquisition (DIA) mode to enhance reproducibility and coverage. Protein expression profiles were visualized via principal component analysis (PCA) to identify inter-group variations.

Differentially expressed proteins (DEPs) were identified based on fold change (FC) ≥ 2 and *p*-value < 0.05 thresholds. Pearson’s correlation coefficient was applied to assess relationships among DEPs. Comparative analyses were conducted for the following groups: U1 vs. U0, U2 vs. U0, and U3 vs. U0. Functional annotation and pathway enrichment were performed using the Gene Ontology (GO) and Kyoto Encyclopedia of Genes and Genomes (KEGG) databases. Comprehensive experimental details are provided in Supplementary S1.

### 2.8 Metabolomic analysis (LC–MS)

After 7 days of uranium (U) exposure, microalgae cells were rapidly collected, immediately frozen in liquid nitrogen, and stored for metabolite extraction. The metabolite extraction protocol was performed as follows: 1 mL of a methanol–water extraction solvent was mixed with 200 μL of chloroform and 20 μL of the internal standard, L-2-chlorophenylalanine. The mixture underwent ultrasonic-assisted extraction, followed by freeze–thaw cycling, centrifugation, vacuum drying, reconstitution in solvent, and vacuum filtration to obtain the final metabolite extract.

Metabolic profiling was conducted using an ultra-performance liquid chromatography (UPLC) system (Nexera, Shimadzu, Japan) coupled to a Q Exactive HF-X high-resolution mass spectrometer (Thermo Fisher Scientific, United States). Data preprocessing, including peak detection, alignment, and normalization, was performed using Progenesis QI v3.0 (Nonlinear Dynamics, Newcastle, UK).

Differentially expressed metabolites (DEMs) were identified via orthogonal partial least squares-discriminant analysis (OPLS-DA) with stringent selection criteria: variable importance in projection (VIP) ≥ 1, *p*-value < 0.05. Significant DEMs were subjected to Kyoto Encyclopedia of Genes and Genomes (KEGG) pathway enrichment analysis to elucidate affected metabolic pathways. Comprehensive experimental details are provided in Supplementary S1.

### 2.9 Data analysis

All statistical analyses were performed using SPSS Statistics (v21.0, IBM, United States). Differences among treatment groups were assessed by one-way analysis of variance (ANOVA), followed by Fisher’s least significant difference (LSD) *post-hoc* test to identify statistically significant differences. A significance threshold of *p* < 0.05 was applied for all comparisons. Data visualization was conducted using Microsoft Excel 2010 (Microsoft, United States), Origin (United States), and OmicShare online tools (https://www.omicshare.com/tools/). Final figure refinement, including layout adjustments and esthetic enhancements, was performed using Adobe Illustrator (United States).

## 3 Results and discussion

### 3.1 Physiological responses of *Ulothrix* sp. to uranium exposure

**Figure 1:**
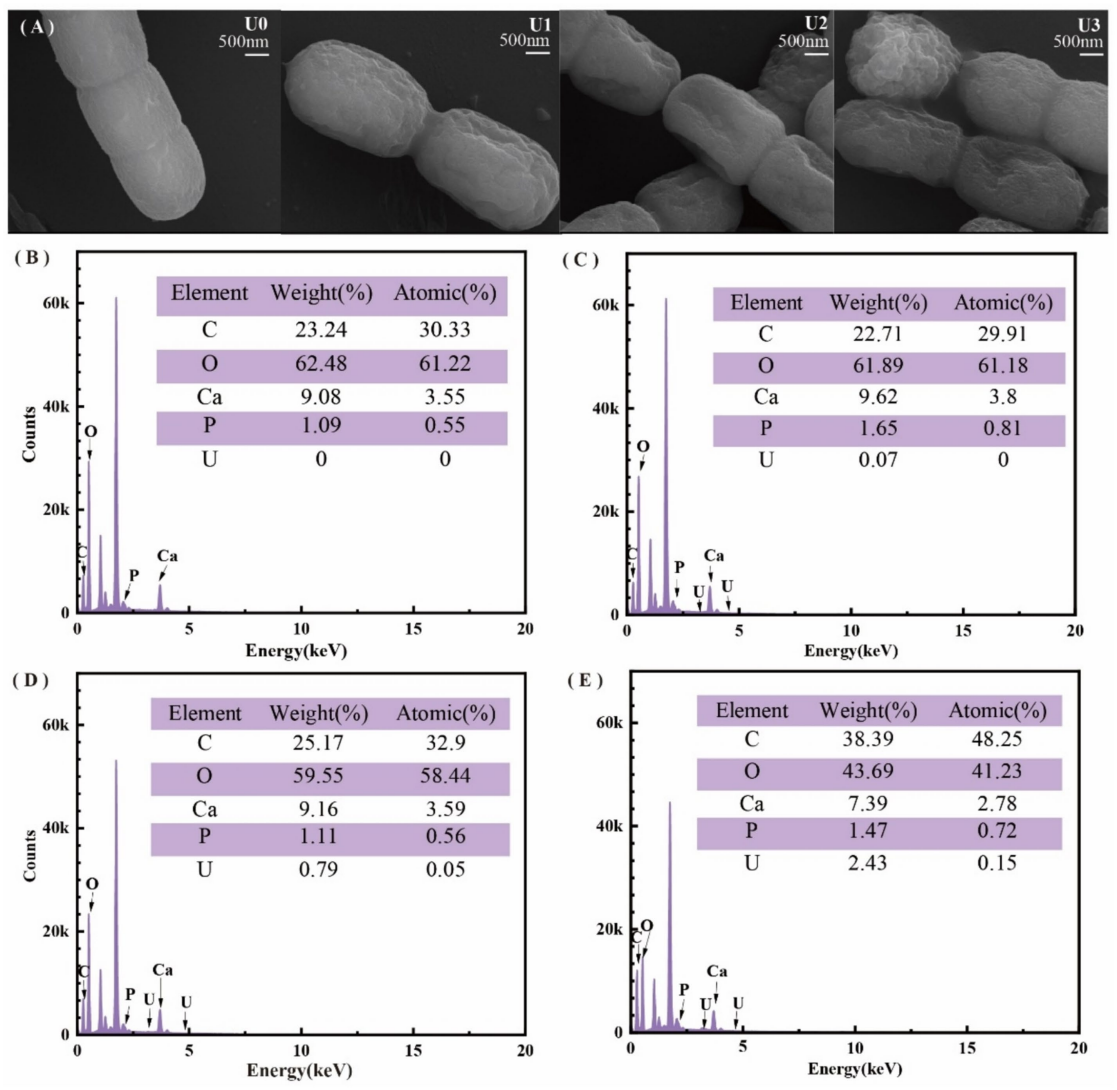
Effects of different uranium treatments on the physiological characteristics of *Ulothrix* sp. **(A)** Scanning electron microscopy (SEM) images. **(B–E)** Energy-dispersive X-ray spectroscopy (EDS) analysis. Data represent the mean ± SD of three independent biological replicates. Different lowercase letters indicate statistically significant differences (*P* < 0.05) among treatment groups at the same sampling time.

The physiological effects of uranium concentrations on *Ulothrix* sp. after 7 days of exposure are presented in Figure 1. SEM observations (Figure 1A) demonstrated that increasing U concentrations induced progressive morphological changes in algal cells, characterized by irregular cell membrane shrinkage hallmark of ion stress-induced deformation. EDS confirmed significant U accumulation on the cell surface (Figures 1B–E), with relative U contents of 0% (control), 0.07, 0.79, and 2.43% in the respective treatment groups (U0–U3). Notably, EDS analysis revealed a correlated distribution pattern between U and phosphorus (P) on the cell surface, suggesting potential mechanisms of bioaccumulation.

FT-IR spectroscopy (Supplementary Figure S1) revealed alterations in surface functional groups; the U1 group spectral profiles closely resembled the U0, indicating minimal perturbation. U2 and U3 concentration groups: Amide band intensification: increased absorption peaks at 1648.0 cm^−1^ (amide I, C=O stretching) and 1545.0 cm^−1^ (amide II, N-H/C-H bending) suggested protein structural modifications. Phosphate group shifts: the P-O stretching vibration peak (U0: 1040.0 cm^−1^) shifted to 1000.6 cm^−1^ post-exposure, implicating direct interactions between uranyl ions (UO₂^2+^) and phosphate-containing biomolecules (e.g., phospholipids, nucleic acids).

These findings collectively demonstrate that uranium exposure induces morphological damage (cell membrane shrinkage), surface U bioaccumulation, and evidence of protein and phosphate group alterations, highlighting mechanisms of uranium toxicity in *Ulothrix* sp.

### 3.2 Growth and photosynthetic impairment in *Ulothrix* sp. under uranium stress

**Figure 2:**
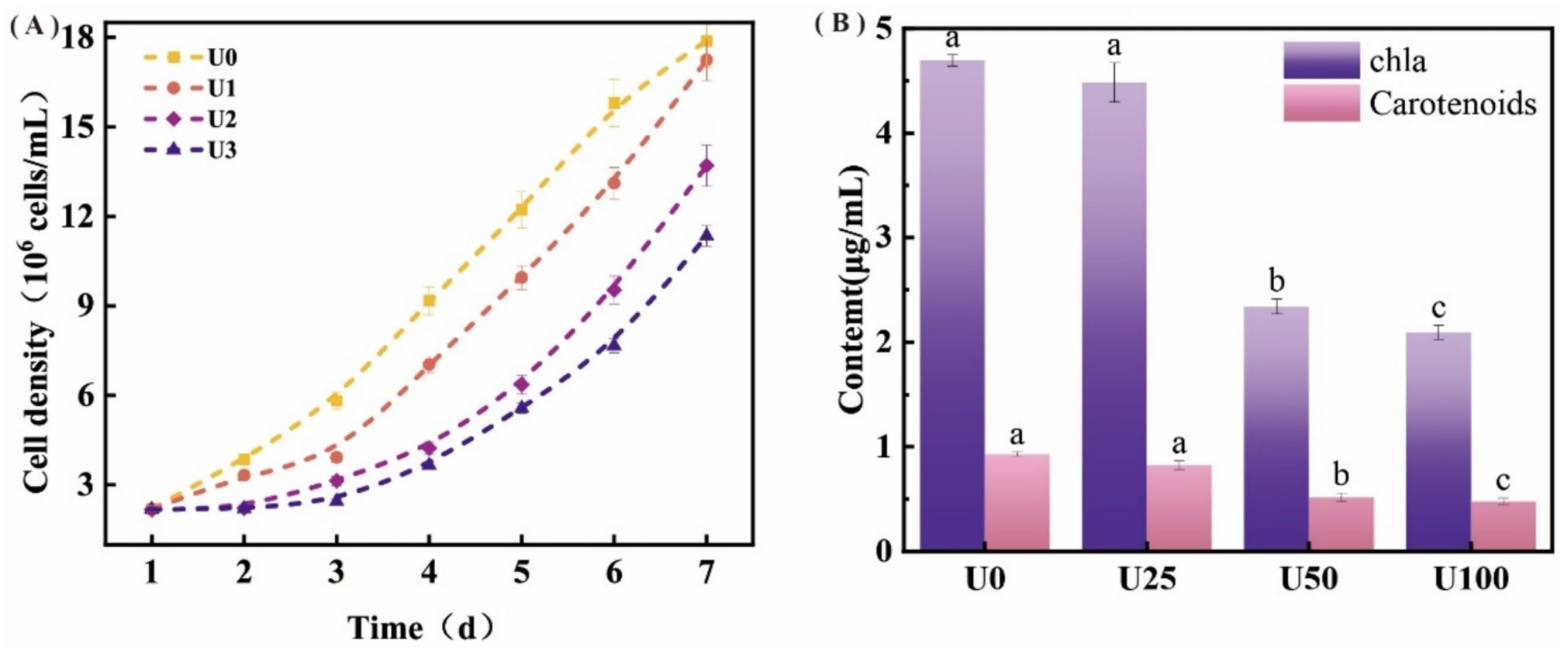
Effects of different uranium treatments on the growth and photosynthesis of *Ulothrix* sp. **(A)** Growth curve of *Ulothrix* sp. under different treatment concentrations. **(B)** Chlorophyll a (Chl a) and carotenoid content after exposure to uranium. Data represent the mean ± SD of three independent biological replicates. Different lowercase letters indicate statistically significant differences (*P* < 0.05) among treatment groups at the same sampling time.

Uranium exposure significantly inhibited the growth of *Ulothrix* sp. with cell density declining in a dose-dependent manner (Figure 2A). Consistent with biomass reduction, Chl *a* and carotenoid contents were significantly reduced by 49–65% and 51–56%, respectively, under U2 and U3 treatment levels (*p* < 0.05), while the low concentration (U1) showed no significant effect (Figure 2B). The photosystem II maximum quantum yield of PSII (Fv/Fm) decreased notably under U2 and U3 treatments, indicating damage to PSII reaction centers. Key fluorescence parameters including absorption (ABS/RC), trapped energy (TRo/RC), and dissipated energy flux per reaction center (DIo/RC) increased significantly by 1.63–2.07, 1.20–2.62, and 1.82–2.01-fold, respectively (Table 1; Supplementary Figure S2). The electron transport flux per reaction center (ETo/RC) also exhibited an increasing trend, suggesting enhanced energy dissipation and reduced electron transport efficiency under U stress.

**Table 1:** Effects of U exposure on kinetics parameters of rapid chlorophyll fluorescence induction in microalgae.

Parameters	Treatment group			
	U0	U1	U2	U3
Fv/Fm	0.297 ± 0.008a	0.294 ± 0.005a	0.217 ± 0.010b	0.187 ± 0.011c
ABS/RC	2.730 ± 0.138c	2.800 ± 0.230c	4.472 ± 0.309b	5.653 ± 0.286a
ETo/RC	0.487 ± 0.013b	0.500 ± 0.042b	0.526 ± 0.032a	0.574 ± 0.168a
TRo/RC	0.809 ± 0.028c	0.822 ± 0.068c	0.972 ± 0.086b	2.119 ± 0.416a
DIo/RC	1.921 ± 0.114c	1.978 ± 0.165c	3.500 ± 0.239b	3.867 ± 0.601a
Pi_Abs	0.235 ± 0.023a	0.231 ± 0.021a	0.074 ± 0.008b	0.085 ± 0.064b

Different lowercase letters indicated significant differences at the 0.05 level (*N* = 3).

Morphological and spectral changes (cell membrane shrinkage, U-P distribution correlation, and FT-IR peak shift) indicate U binding to cell surface components, such as peptidoglycan, polysaccharides, and phospholipids, consistent with previous reports (Dutta et al., 2022; Liu et al., 2022; Yu et al., 2022). This binding may lead to the precipitation and accumulation of uranium on the cell surface, thereby disrupting membrane integrity and altering membrane permeability (Kolhe et al., 2020). Under uranium stress, although TRo/RC increased, the efficiency of energy utilization for photochemistry (reflected by decreased Fv/Fm and altered ETo/RC) declined. Consequently, excess light energy was predominantly dissipated as heat via non-photochemical quenching (NPQ), resulting in a significant rise in DIo/RC. This enhanced energy dissipation likely constitutes an adaptive protective mechanism that mitigates oxidative damage to the PSII complex caused by excessive excitation energy (Li et al., 2022). However, under medium and high U exposure, this mechanism was insufficient to fully counteract the damage, ultimately leading to photosynthetic pigment reduction and growth inhibition (Figures 1F,G).

### 3.3 Effect of uranium exposure on elemental uptake in *Ulothrix* sp.

**Figure 3:**
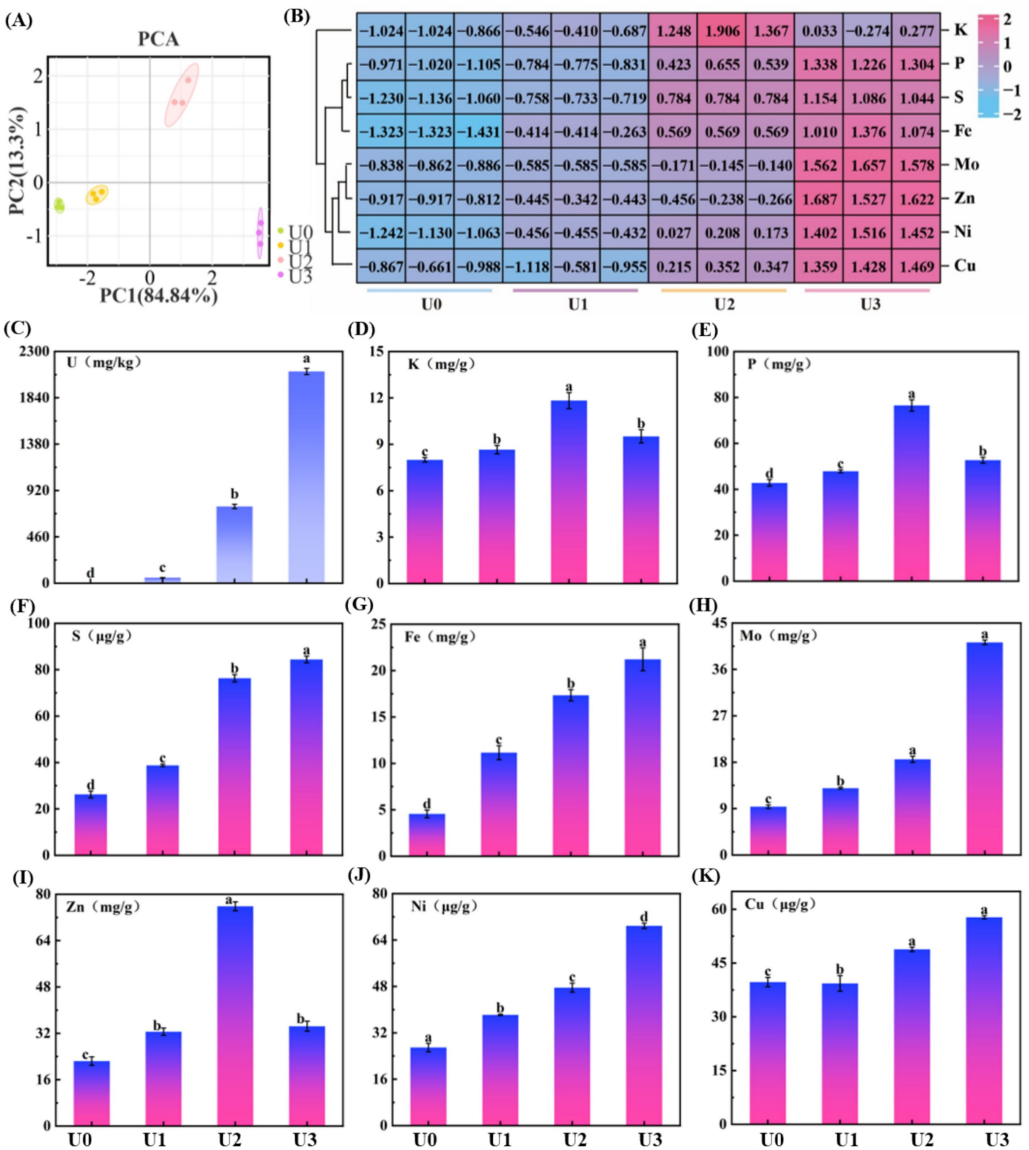
Effects of uranium exposure on elemental content in *Ulothrix* sp. **(A)** Principal component analysis (PCA). **(B)** Heatmap of mineral elements. **(C)** Uranium enrichment characteristics. **(D–K)** Concentrations of mineral elements. Data represent the mean ± SD of three independent biological replicates. Different lowercase letters indicate statistically significant differences (*P* < 0.05) among treatment groups at the same sampling time.

Mineral elements are essential micronutrients for organismal growth, participating in numerous biological functions, such as photosynthesis, respiration, and enzymatic reactions. Principal component analysis (PCA) results (Figure 3A) showed that samples from different U treatment groups (U0, U1, U2, U3) exhibited distinct separation trends in the PCA plot, indicating that U exposure significantly altered the mineral element composition within *Ulothrix* sp. (Figure 3B). *Ulothrix* sp. exhibited significant uranium enrichment capability. The uranium content in the microalgal biomass (dry weight) of the treatment groups (U1, U2, U3) was 54 mg/kg DW, 761 mg/kg DW, and 2,100 mg/kg DW, respectively (Figure 3C), and the enrichment level was positively correlated with the exposure dose. The U accumulation capacity of *Ulothrix* sp. (2,100 mg/kg DW) exceeds values reported for many common microalgae, such as *Chlorella vulgaris* [typically <600 mg/kg DW under comparable conditions (Camille et al., 2024)]. The content of mineral elements was also significantly affected by U exposure (Figures 3D–K). With increasing U exposure concentration, the contents of S, Fe, Ni, Cu, and Mo showed a significant increasing trend. The changes in P and zinc Zn contents exhibited a different pattern. They initially increased and then decreased with increasing exposure concentration, showing a significant reduction in the U3 group.

The results indicate that U exposure induced a reconstruction of the mineral element profile in *Ulothrix* sp. The underlying mechanism may involve competitive absorption between elements and regulation of transport. For example, this study found a decrease in P content in the high-concentration (U3) treatment group (Figure 3E), which corroborates the characteristic peak shift of the phosphate group observed in FT-IR (Supplementary Figure S1), suggesting competitive inhibition or shared transport pathways between uranium (U) and phosphate (PO₄^3−^) (Schilz et al., 2022). Uranyl ions (UO₂^2+^) may bind to phosphate groups on the cell surface or competitively bind to phosphate transporters, thereby interfering with normal phosphorus uptake. Previous studies have indicated that uranium can compete with Zn^2+^ for the same divalent cation transporters (Hayek et al., 2018). Furthermore, the decrease in Zn content observed in the U3 group (Figure 3I) may result from the competitive inhibition of Zn uptake by U, leading to reduced Zn levels.

The cell membrane is a critical interface for substance transport, including ion translocation (Nguyen et al., 2022). Morphological damage to the cell membrane induced by U exposure (Figure 1A) compromises membrane integrity and consequently affects the function of membrane transport proteins and the efficiency of ion transmembrane transport. That may be one of the crucial reasons for the disordered absorption of multiple mineral elements.

### 3.4 Integrated antioxidant defense mechanisms under uranium stress

**Figure 4:**
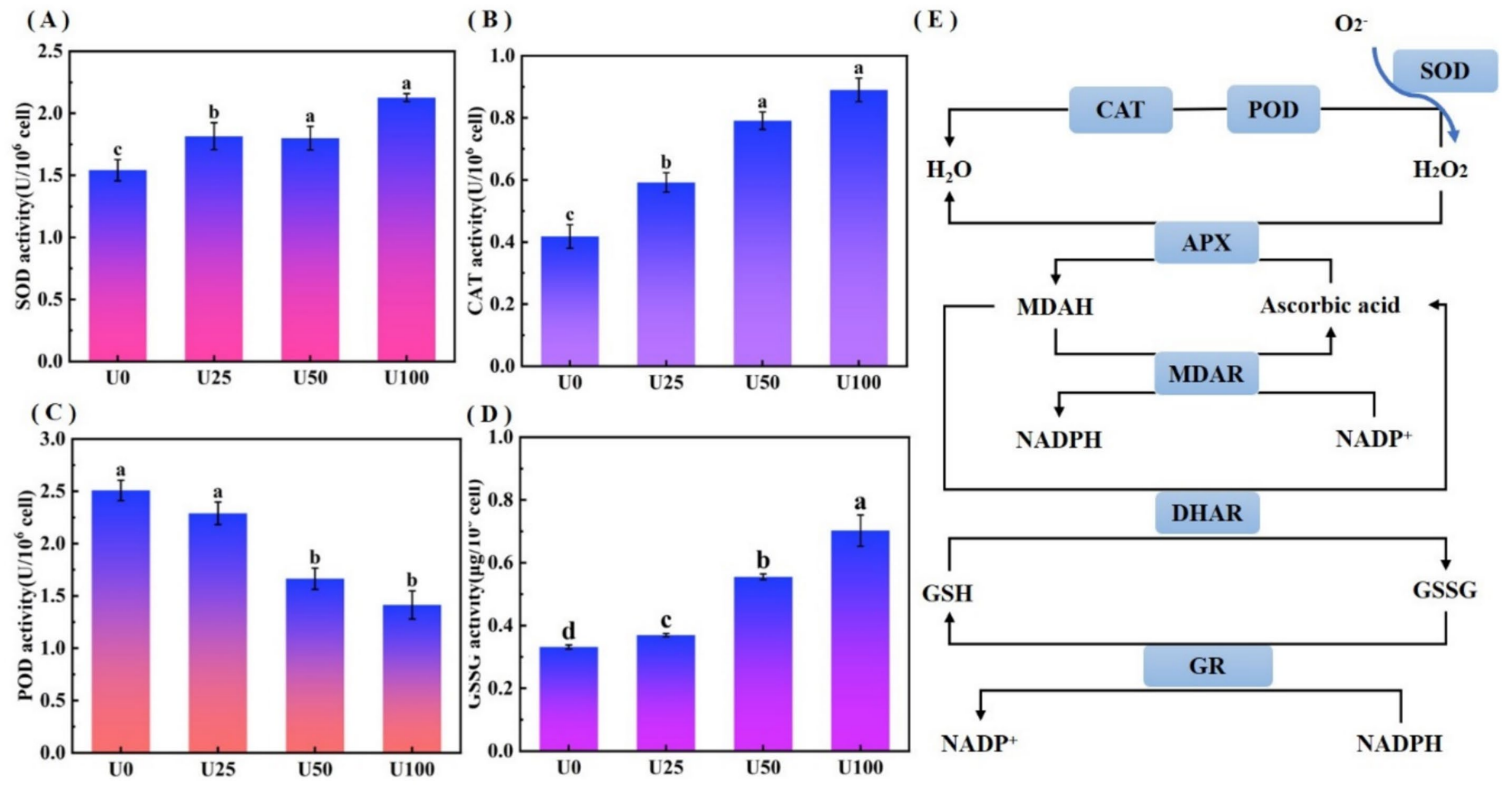
Oxidative stress analysis of *Ulothrix* sp. under uranium stress. **(A)** SOD activity. **(B)** CAT activity. **(C)** POD activity. **(D)** Oxidized glutathione (GSSG) content. **(E)** Schematic of oxidative stress response network. Values represent the mean of three independent biological replicates. Different lowercase letters indicate statistically significant differences (*P* < 0.05) among treatment groups at the same sampling time.

Uranium stress induced reactive oxygen species (ROS) accumulation, activating the antioxidant defense system of *Ulothrix* sp. The oxidative stress analysis is shown in Figure 4. Compared to the U0, SOD activity in all treatment groups (U1, U2, U3) was significantly upregulated (*p* < 0.05) (Figure 4A), exhibiting a dose-dependent enhancement trend. SOD is the first line of defense for scavenging superoxide anion radicals (O₂•^−^). The increase in its activity indicates that *Ulothrix* sp. effectively enhanced its capacity to convert O₂•^−^ into hydrogen peroxide (H₂O₂). CAT activity also showed a significant upregulation trend (*p* < 0.05) (Figure 4B). The enhancement of CAT activity indicates that *Ulothrix* sp. improved its ability to directly decompose and scavenge H₂O₂ (catalyzing the decomposition of H₂O₂ into H₂O and O₂). POD activity in all U exposure treatment groups (U1, U2, U3) was significantly lower than that in the U0 group (*p* < 0.05), and showed a significant decreasing trend with increasing U concentration (Figure 4C). U exposure led to a significant increase (*p* < 0.05) in the content of oxidized glutathione (GSSG) in Ulothrix sp. cells (Figure 4D). In the U3 group, the GSSG content reached 2.2 times that of the control.

The activated antioxidant defense system effectively mitigated uranium-induced intracellular ROS accumulation, forming a critical physiological basis for *Ulothrix* sp.’s adaptive response to uranium stress (Figure 4E). This integrated response involves sequential enzymatic reactions: SOD catalyzes the dismutation of superoxide anion radicals (O₂•^−^) into hydrogen peroxide (H₂O₂) and molecular oxygen (O₂), while CAT further decomposes H₂O₂ into water (H₂O) and O₂ (Moenne et al., 2020). Concurrent with the activation of antioxidant enzymes, significant GSSG accumulation (Figure 4D) served as a key biomarker of oxidative stress. This GSSG increase is typically accompanied by reduced glutathione (GSH) depletion, thereby disrupting the cellular redox equilibrium. To restore redox homeostasis, GSSG is recycled into GSH through glutathione reductase (GR)-mediated reactions (Rola et al., 2022). The GSH/GSSG redox pair constitutes the core component of the cellular antioxidant system, directly modulating resistance to heavy metal-induced oxidative damage (Kizilbay and Karaman, 2022).

### 3.5 Effect of uranium exposure on the proteome of *Ulothrix* sp.

Proteomic analysis identified a total of 1,412 proteins, with 3,403 peptides ranging in length from 7 to 20 amino acids (Supplementary Figures S3A–C). Principal component analysis (PCA) revealed significant differences in protein expression profiles among Ulothrix sp. treatment groups (Supplementary Figure S2D). Pearson correlation coefficient analysis further confirmed high reproducibility within groups and distinct clustering between groups (Supplementary Figure S3E), indicating that U exposure reshaped the protein expression profile of *Ulothrix* sp. Analysis of differentially expressed proteins (DEPs) between groups showed that in the U1 group, 595 DEPs were screened (447 upregulated, 148 downregulated). In the U2 group, 722 DEPs were screened (569 upregulated, 153 downregulated). In the U3 group, 255 DEPs were screened (121 upregulated, 134 downregulated) (Supplementary Figure S4A). The decrease in DEPs at U3 likely reflects partial metabolic shutdown and loss of cellular viability, as supported by reduced Fv/Fm and elevated oxidative damage markers. Subcellular localization prediction indicated that DEPs were primarily distributed in the Cytoplasm, accounting for over 50% in all groups (Supplementary Figure S4B).

GO functional enrichment results are shown in Supplementary Figure S5. In the U1 group, the highest number of proteins in the biological process was “Carbohydrate metabolic process” (21), and the highest numbers in the cellular components were “Cytoplasm” (99) and “Cytosol” (91). In the U2 group, the top biological processes were “Translation” (28) and “Carbohydrate metabolic process” (25). In the U3 group, the top biological processes were “Translation” (11) and “Carbohydrate metabolic process” (8). The top cellular component was “Cytosol” (25). “Translation” and “Carbohydrate metabolic process” were commonly enriched pathways across all concentration groups.

**Figure 5:**
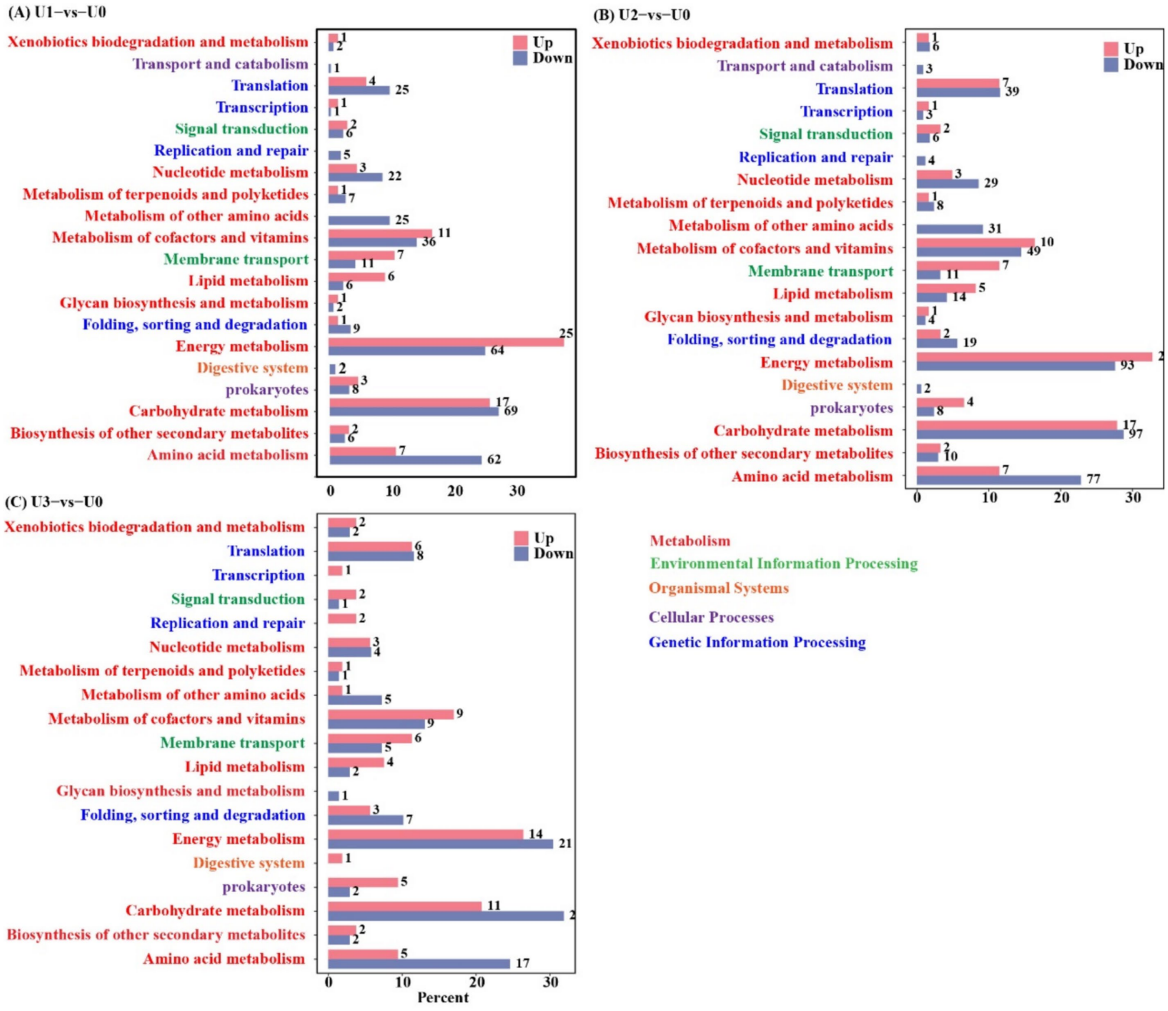
KEGG annotation analysis of differentially expressed proteins (DEPs). Detailed metabolic pathways of DEPs are provided in Supplementary Tables S1–S3. **(A-C)** Between-group comparisons included: U1 vs U0; U2 vs U0 and U3 vs U0.

**Figure 6:**
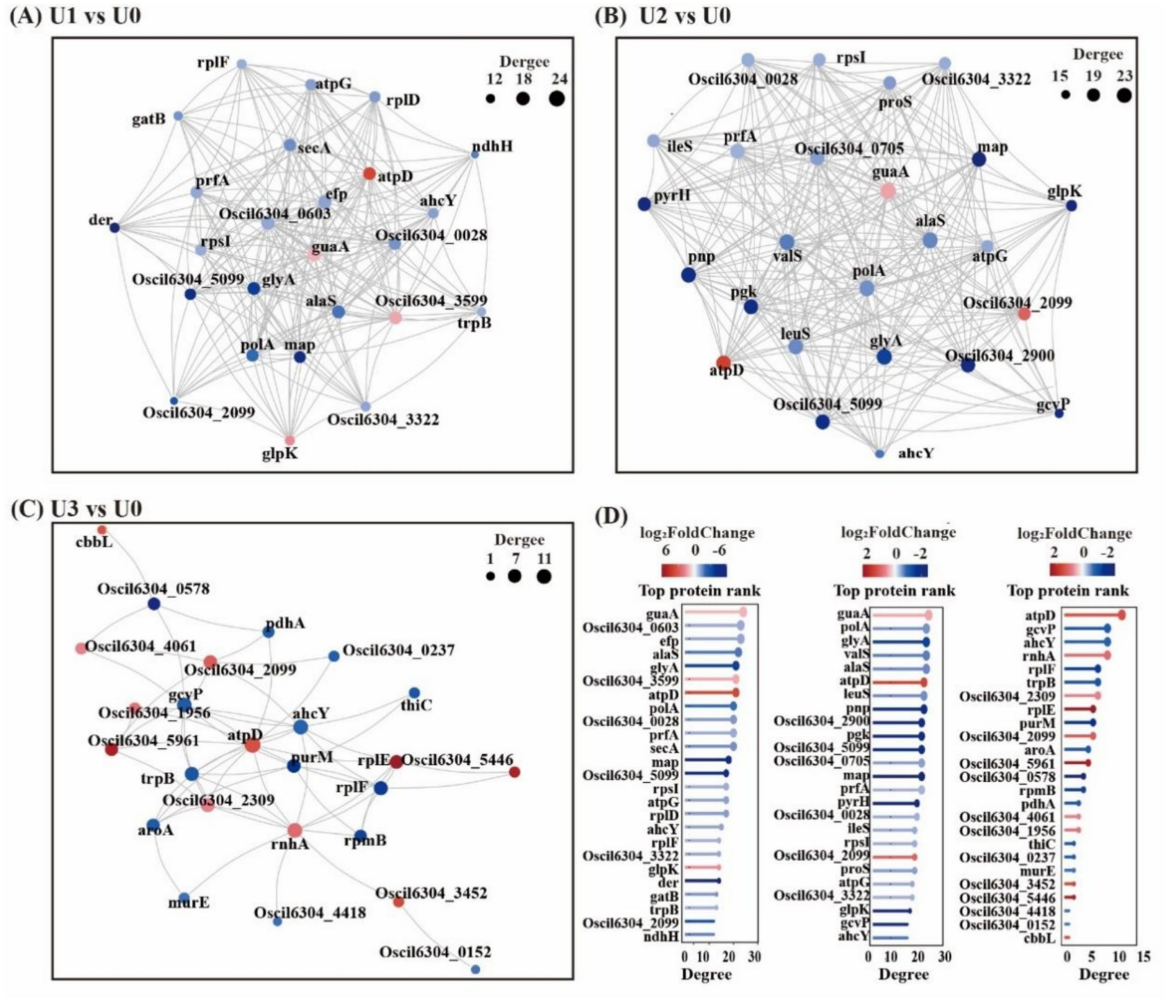
Protein–protein interaction (PPI) network (Top25). Circles represent DEPs/genes (red: up-regulated; blue: down-regulated). Circle size indicates the degree of connectivity (larger = higher connectivity). **(A-C)** Between-group comparisons included: U1 vs U0, U2 vs U0 and U3 vs U0. **(D)** Bar chart of Top25 connectivity proteins and shared legends across groups. Details in Supplementary Tables S4–S6.

KEGG pathway analysis indicated that U stress primarily affected protein expression in pathways related to “Carbohydrate metabolism,” “Amino acid metabolism,” and “Energy” (Figure 5). Protein–protein interaction (PPI) network topology analysis of key nodes revealed that the ATP synthase beta subunit (atpD) occupied a central position in the network across all U1-U3 groups (Figure 6), with atpD exhibiting sustained high expression (fold change: 14.5 → 18.9 → 4.0).

Overall, under low concentration stress, the number of DEPs increased, indicating that cells actively responded to stress by enhancing translation and metabolism. However, under high-concentration stress, the total number of DEPs sharply decreased. Cells conserve energy by suppressing non-essential biological processes and reallocating resources toward critical defense pathways (Judith et al., 2023). This study found that the U stress response focused on the cytosol compartment, with “Translation” and “Carbohydrate metabolism” serving as core regulatory pathways. These pathways synergistically maintain protein homeostasis and energy balance in response to stress. The sustained high expression of atpD, a key component of ATP synthase, indicates that U stress-induced reactive oxygen species (ROS) burst forced cells to accelerate ATP synthesis to support energy-consuming processes such as antioxidant defense and ion transport.

### 3.6 Effect of uranium stress on the metabolic network

**Figure 7:**
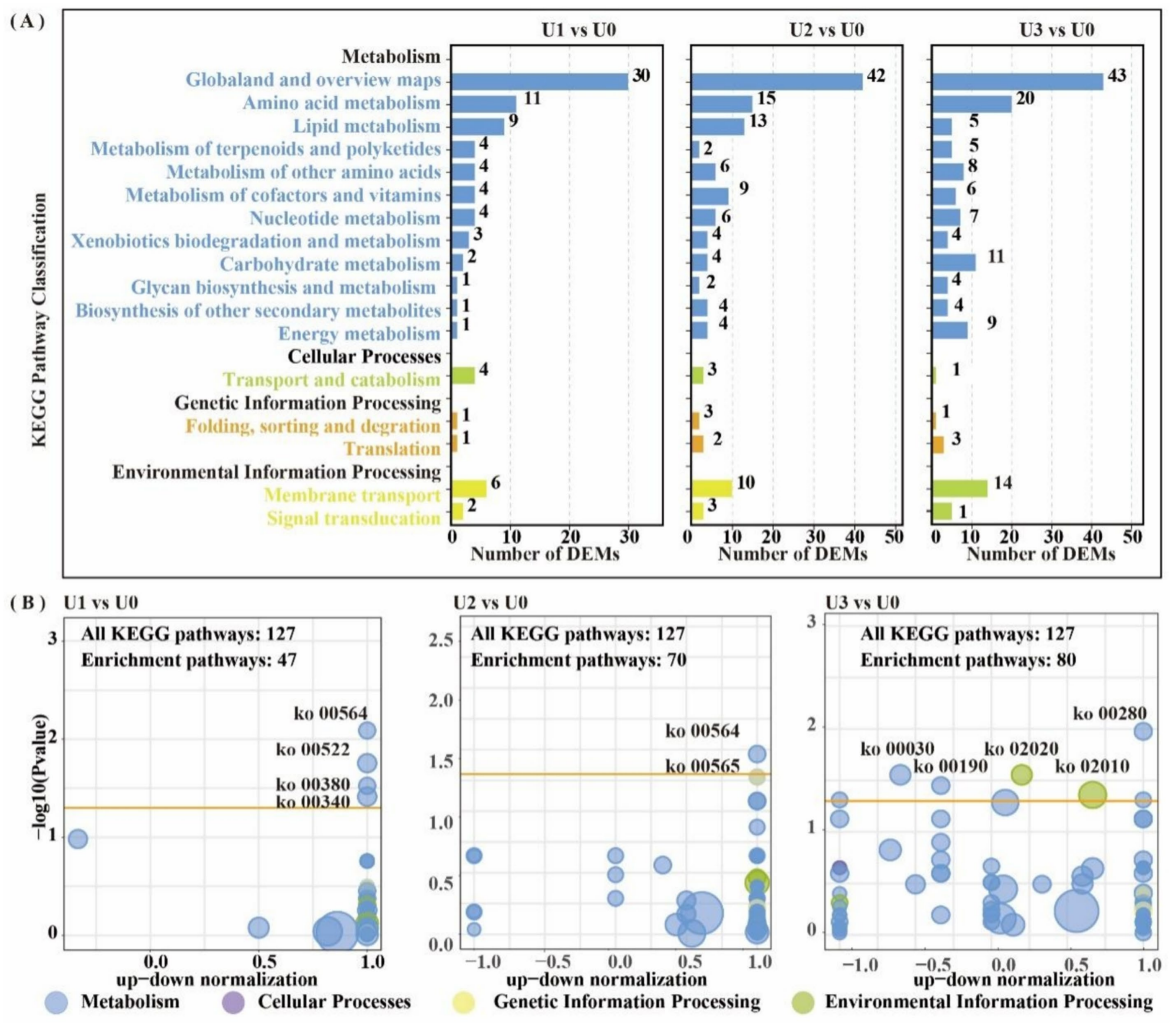
KEGG enrichment pathway analysis of differentially expressed metabolites (DEMs). DEM-associated pathway details are in Supplementary Table S7. **(A)** KEGG pathway classification in all group comparisons; **(B)** Metabolic pathway interaction network analysis in U1 vs U0, U2 vs U0 and U3 vs U0.

**Figure 8:**
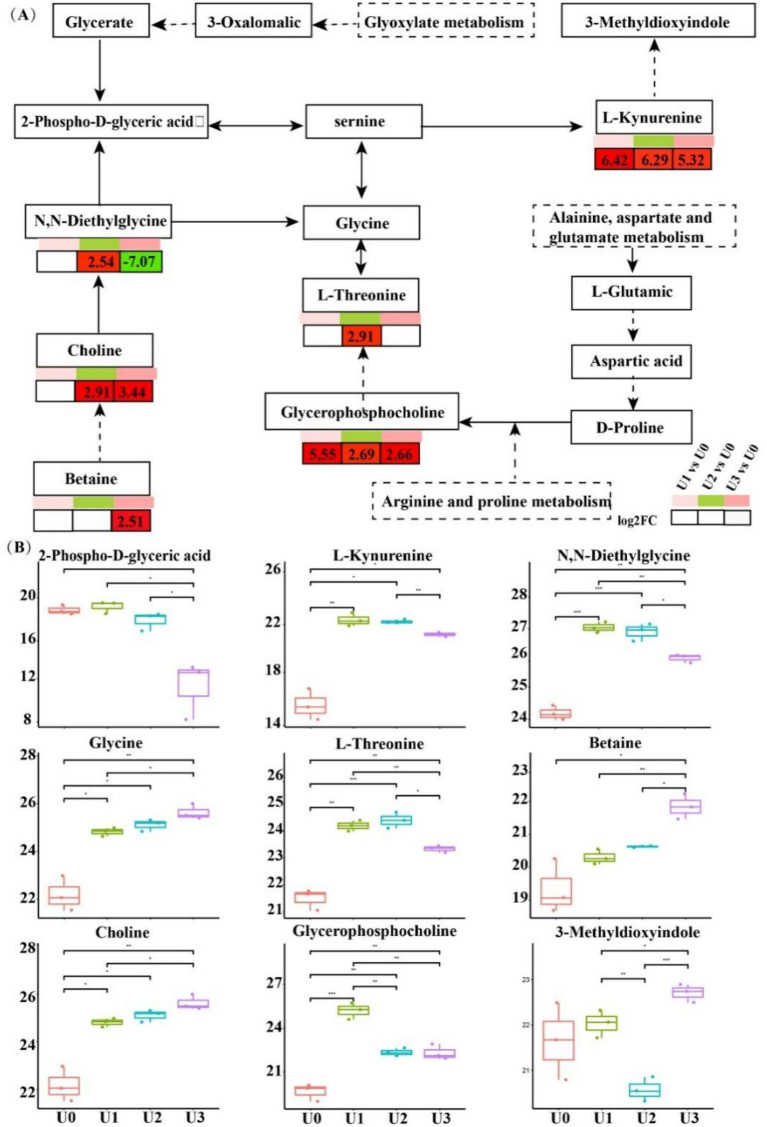
Alterations of metabolites related to Gly-Ser-Thr metabolism pathway. *, ** indicated that the *T* test had a significant difference at 0.05 and 0.01 levels. **(A)** Gly-Ser-Thr metabolism pathway. **(B)** Differential expression of key metabolites.

LC–MS untargeted metabolomics identified a total of 2,969 metabolites. PCA results showed clear separation between treatment groups and the control group (Supplementary Figure S6). In the U1 vs. U0, 928 DEMs were identified (Significant up: 23, Significant down: 905). In the U2 vs. U0, 807 DEMs were identified (Significant up: 99, Significant down: 708). In the U3 vs. U0, 734 DEMs were identified (Significant up: 51, Significant down: 683) (Supplementary Figure S7). KEGG metabolic pathway analysis (Figure 7A) revealed that DEMs were primarily concentrated in the “Amino acid metabolism” and “Lipid metabolism” pathways. In the U3 group, DEMs were mainly enriched in “Amino acid metabolism” and “Carbohydrate metabolism.” Enriched pathway analysis (Figure 7B) showed that in the U1 and U2 exposure groups, differential metabolites were significantly enriched in the “Glycerophospholipid metabolism” (ko00564) pathway. In the U3 group, differential metabolites were significantly enriched in “Valine, leucine and isoleucine degradation” (ko00280), “Pentose phosphate pathway” (ko00030), and “Oxidative phosphorylation” (ko00190). Further analysis of the Glycerophospholipid metabolism (ko00564) pathway (Figure 8) revealed that Glycine and L-Threonine are closely associated with glycerophospholipid metabolism.

The enrichment of glycerophospholipid metabolism in the low and medium treatment groups (U1/U2) indicates that *Ulothrix* sp. adjusts its metabolism through associated changes in Glycine and L-threonine to support this pathway. In the group U3, the pentose phosphate pathway generates NADPH to support antioxidant defense, while the oxidative phosphorylation pathway efficiently synthesizes ATP. Dimethylglycine (DMG) can effectively scavenge biological free radicals (Aon et al., 2020); its significant attenuation in the U3 group is directly linked to the inhibition of algal cell growth (Figure 1A). The upregulation of metabolites such as amino acids indicates that threonine, aspartic acid, and others alleviate oxidative damage by scavenging ROS and maintaining the TCA cycle, collectively forming a stress defense barrier together with lipid metabolism (Li et al., 2023; Zhang et al., 2025b).

### 3.7 Effect of uranium exposure on the fundamental energy cycle of *Ulothrix* sp.

**Figure 9:**
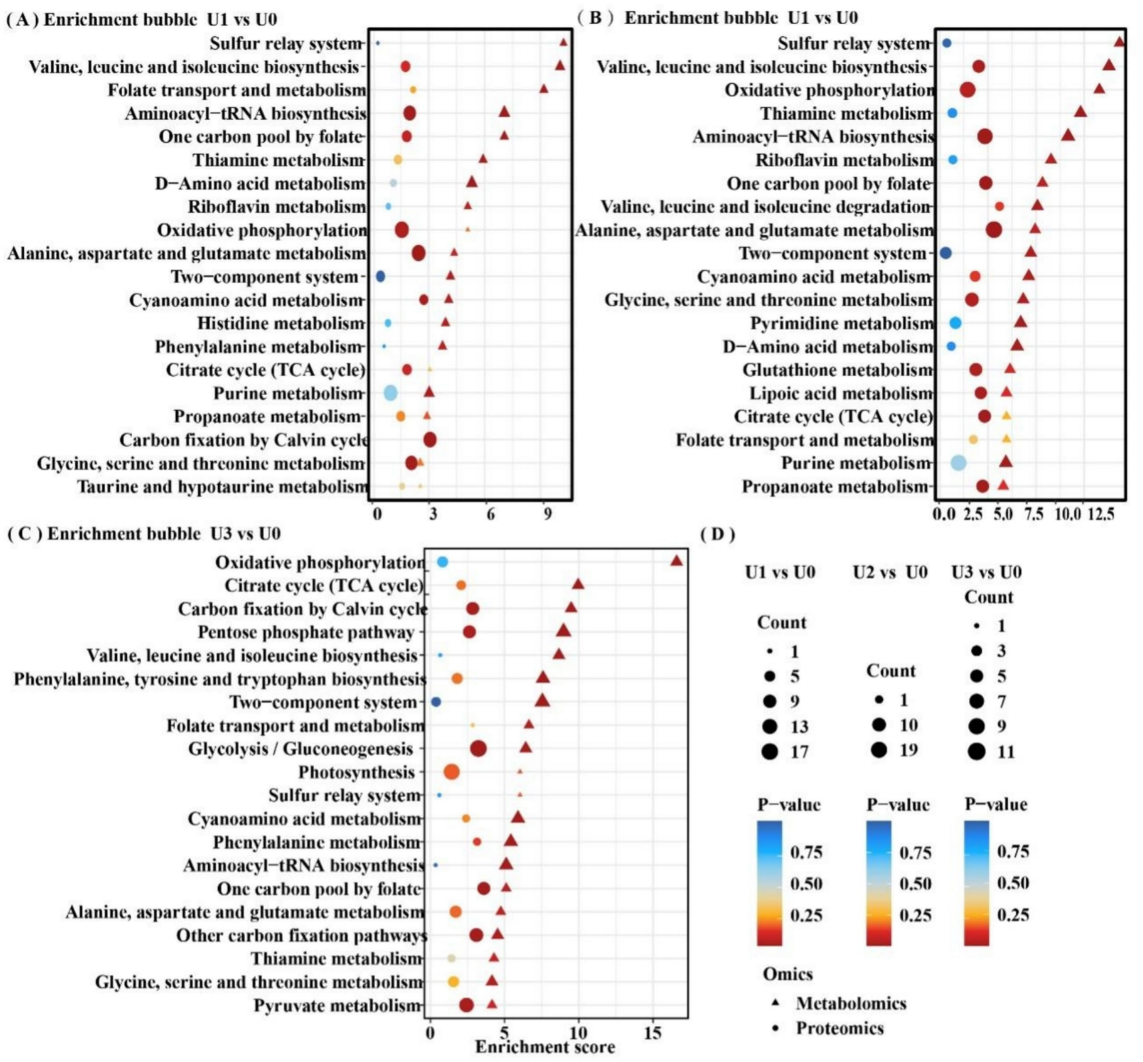
Shared differentially enriched pathway analysis of proteins and metabolites. Detailed shared pathways are in Supplementary Table S8. **(A-C)** Between-group comparisons included: U1 vs U0, U2 vs U0 and U3 vs U0. **(D)** Common legend annotation.

**Figure 10:**
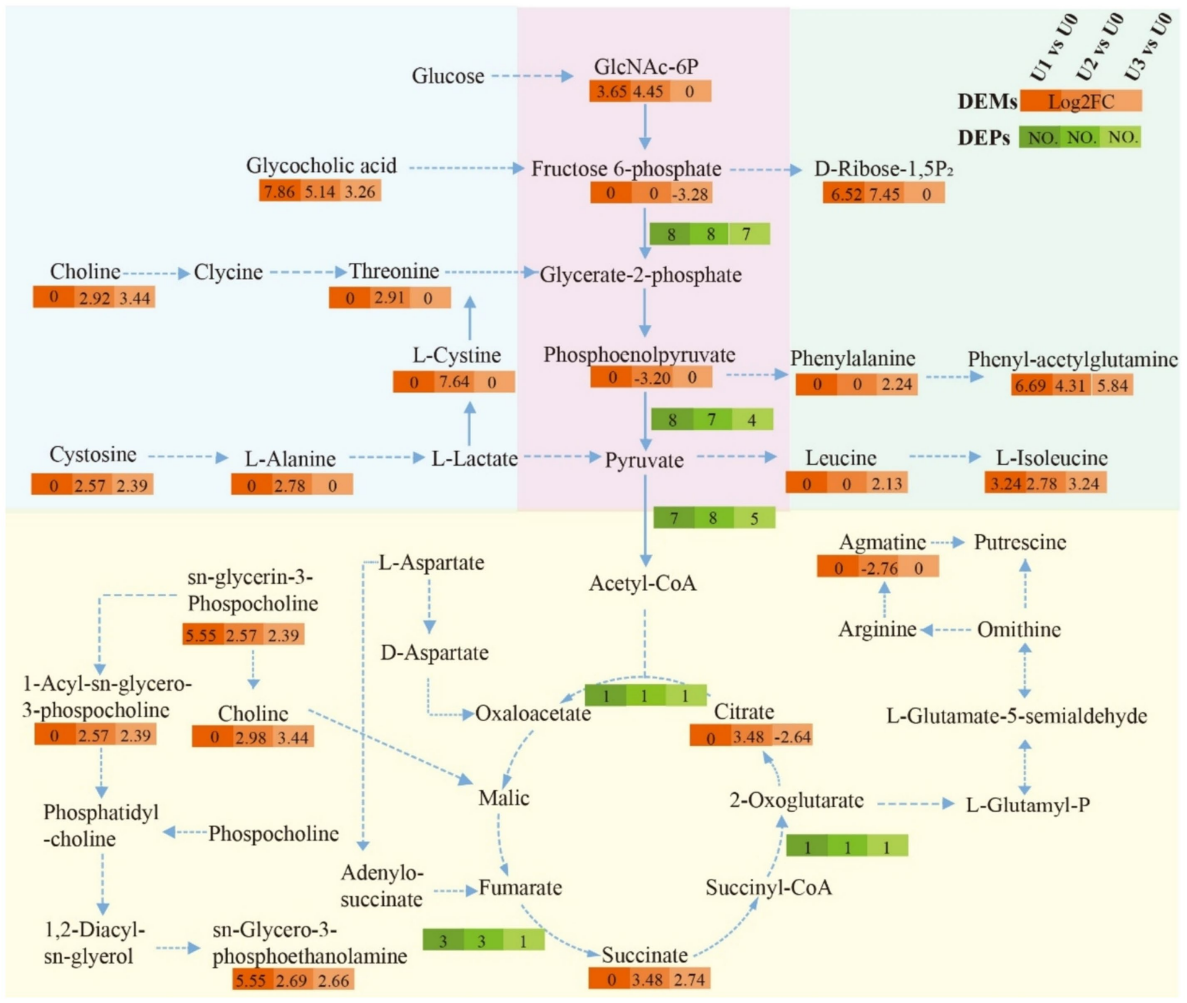
Impact of uranium exposure on the fundamental energy cycle of *Ulothrix* sp. Solid arrows indicate direct pathways; dashed arrows indicate indirect pathways.

Based on the joint analysis of DEPs and DEMs, a bubble plot was constructed to visualize the shared pathways (Figure 9), aiming to systematically decipher the molecular response mechanisms of *Ulothrix* sp. under uranium stress. Shared pathways included the TCA cycle and Oxidative phosphorylation. Based on these findings, a schematic diagram of the fundamental energy metabolism pathway in Ulothrix sp. was constructed (Figure 10). Within the TCA cycle, metabolites associated with malate (such as choline) were upregulated. Succinate was upregulated, while glycerophospholipid and phenylalanine were significantly upregulated. Citrate was downregulated under high-concentration exposure. The upregulation of malate and succinate drives an increase in TCA cycle flux, providing reducing power (NADH/FADH₂) for ATP synthesis (via oxidative phosphorylation) (Qu et al., 2021). The downregulation of citrate (the initial metabolite of the TCA cycle) in the U3 group indicates that high-concentration uranium directly inhibits respiration, leading to impaired function of this energy hub. The sustained upregulation of glycerophospholipid suggests that *Ulothrix* sp. responds to oxidative stress by enhancing membrane lipid synthesis to maintain cell membrane integrity and reduce lipid peroxidation damage. Previous studies have shown that lipid accumulation is a survival strategy for microalgae under stress conditions, protecting cells from oxidative stress (Zhang et al., 2023).

The results indicate that the acceleration of the glycolytic pathway and TCA cycle can provide energy to overcome the inhibition of cellular energy production caused by stress, demonstrating that the fundamental energy cycle of *Ulothrix* sp. plays a key role in resisting adversity.

## 4 Conclusion

*Ulothrix* sp. exhibits significant uranium enrichment capability (reaching 2,100 mg/kg DW under 400 μmol/L exposure), and its U accumulation increases in a dose-dependent manner with exposure concentration. High-concentration U exposure inhibited the growth of *Ulothrix* sp., significantly interfered with the synthesis of chlorophyll and carotenoids, and had a marked effect on photosynthetic parameters. It suppressed light energy capture efficiency and impaired photosystem II (PSII). Uranium exposure damaged the microalgal cell structure and disrupted ion transport, with Zn and P exhibiting competitive inhibition against U. Uranium exposure induces a burst of reactive oxygen species (ROS), triggering the cells to activate their antioxidant defense systems. Integrated proteomic and metabolomic analysis revealed that under low concentrations, protein synthesis was active, supporting the stress response. Under high-concentration stress, energy metabolism was remodeled: key tricarboxylic acid (TCA) cycle metabolites (succinate, malate) were upregulated, enhancing electron transport efficiency to compensate for photosynthetic damage. Unlike many unicellular algae, *Ulothrix* sp. demonstrates a multi-faceted adaptive strategy under U stress, including the activation of the Gly-Ser-Thr metabolic pathway, synergistic upregulation of SOD/CAT with GSSG accumulation, and sustained energy metabolism via TCA cycle remodeling. These key adaptive strategies collectively enhance its tolerance under high U conditions.

## Data Availability

The original contributions presented in the study are included in the article/Supplementary material, further inquiries can be directed to the corresponding authors.
