# Supplementary material for: Integrated morphological, proteomic and metabolomic analyses reveal response mechanisms of microalgae under uranium exposure
Source: Front Microbiol. 2025 Oct 20;16:1679056. doi: 10.3389/fmicb.2025.1679056 (PMC12581144; doi:10.3389/fmicb.2025.1679056)
Supplement: Supplementary file 2 [file Data_Sheet_2.doc]

**2. Materials and Methods**

**2.8 Protein regulatory factor:**

After incubation for 12 h, the bacterial precipitate was subjected to proteome analysis, and the sample protein was qualitatively and quantitatively analyzed using the Tandem Mass Tags (TMT) in vitro labeling technique. The basic steps included protein extraction, SDS-PAGE protein detection, protease digestion, TMT labeling, high pH liquid chromatographic separation, mass spectrometry, and a database search (M et al., 2018). Reliable proteins were screened based on Score Sequest HT > 0 and unique peptide ≥ 1. The differences in protein expression between groups were visualized by principal component analysis (PCA). The screening threshold of differentially expressed proteins (DEPs) was FC ≥ 2 and p-value < 0.05. The Pearson algorithm was used to calculate the correlation between DEPs. Based on the KEGG and GO databases, the annotation information for the DEPs was extracted, and their protein functions were mined (Kiran et al., 2022).

**2.9** **LC-MS analytical method**

Sample preparation: 1 mL of methanol: water (4/1, vol/vol) were added to each sample, then transformed to a 4 mL glass vial. 200 μL of chloroform were added to each aliquot, dispersing sample by pipette. Using ultrasonic homogenizer to breaking up the cells for 3 min at 500 w. All of the mixtures of each sample were transferred to 1.5 mL Eppendorf tubes, 20 μL of L-2-chlorophenylalanine (0.3 mg/mL) dissolved in methanol as internal standard，then extracted by ultrasonication for 20 min in ice-water bath. The extract was centrifuged at 4°C (13,000 rpm) for 10 min . 1 mL of supernatant in a glass vial was dried in a freeze concentration centrifugal dryer . 300 μL mixture of methanol and water (1/4, vol/vol) were added to each sample, samples vortexed for 30 s, extracted by ultrasonic for 3 min in ice-water bat, then placed at -20°C for 2 h. Samples were centrifuged at 4°C (13,000 rpm) for 10 min. The supernatants (150 μL) from each tube were collected using crystal syringes, filtered through 0.22 μm microfilters and transferred to LC vials. The vials were stored at -80°C until LC -MS analysis. QC samples were prepared by mixing aliquot of the all samples to be a pooled sample.

LC-MS analysis conditions：A Nexera UPLC system (Shimadzu Corporation, Japan) coupled with Q-Exactive quadrupole-Orbitrap mass spectrometer equipped with heated electrospray ionization(ESI) source (Thermo Fisher Scientific, Waltham, MA, USA) was used to analyze the metabolic profiling in both ESI positive and ESI negative ion modes. An ACQUITY UPLC HSS T3 column (1.8 μm, 2.1 × 100 mm) were employed in both positive and negative modes. The binary gradient elution system consisted of (A) water (containing 0.1 % formic acid, v/v) and (B) acetonitrile (containing 0.1 % formic acid, v/v) and separation was achieved using the following gradient: 0 min, 5% B; 2min, 5% B; 4min, 25% B; 8min, 50% B; 10min, 80% B; 14min, 100% B; 15 min, 100% B; 15.1 min, 5% and 16 min, 5%B. The flow rate was 0.35 mL/min and column temperature was 45 ℃. All the samples were kept at 4℃ during the analysis. The injection volume was 2 L.

The mass range was from m/z 100 to 1,200. The resolution was set at 70,000 for the full MS scans and 17,500 for HCD MS/MS scans. The Collision energy was set at 10, 20 and 40 eV. The mass spectrometer operated as follows: spray voltage, 3,500 V (+) and 3,500 V (−); sheath gas flow rate, 40 arbitrary units(+) and 35 arbitrary units(-); auxiliary gas flow rate, 10 arbitrary units(+) and 8 arbitrary units(-); capillary temperature, 320°C.

The QCs were injected at regular intervals (every 3 samples) throughout the analytical run to provide a set of data from which repeatability can be assessed.

**3.1 Physiological Responses of** ***Ulothrix* sp. to Uranium Exposure**


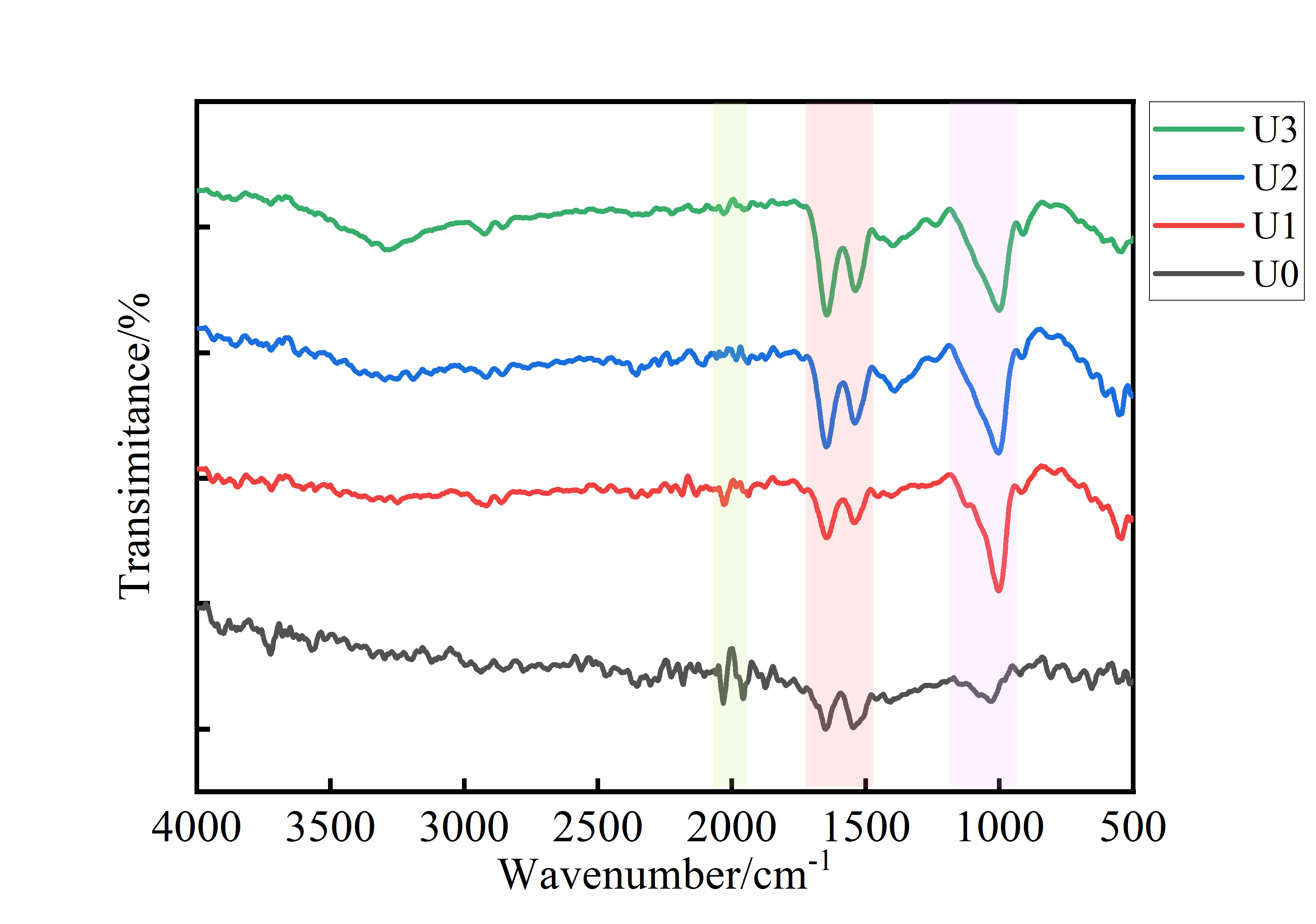


**Fig. S1.** FT-IR analysis of *Ulothrix* sp. to Uranium Exposure

**3.5 Effect of Uranium Exposure on the Proteome of *Ulothrix* sp.**


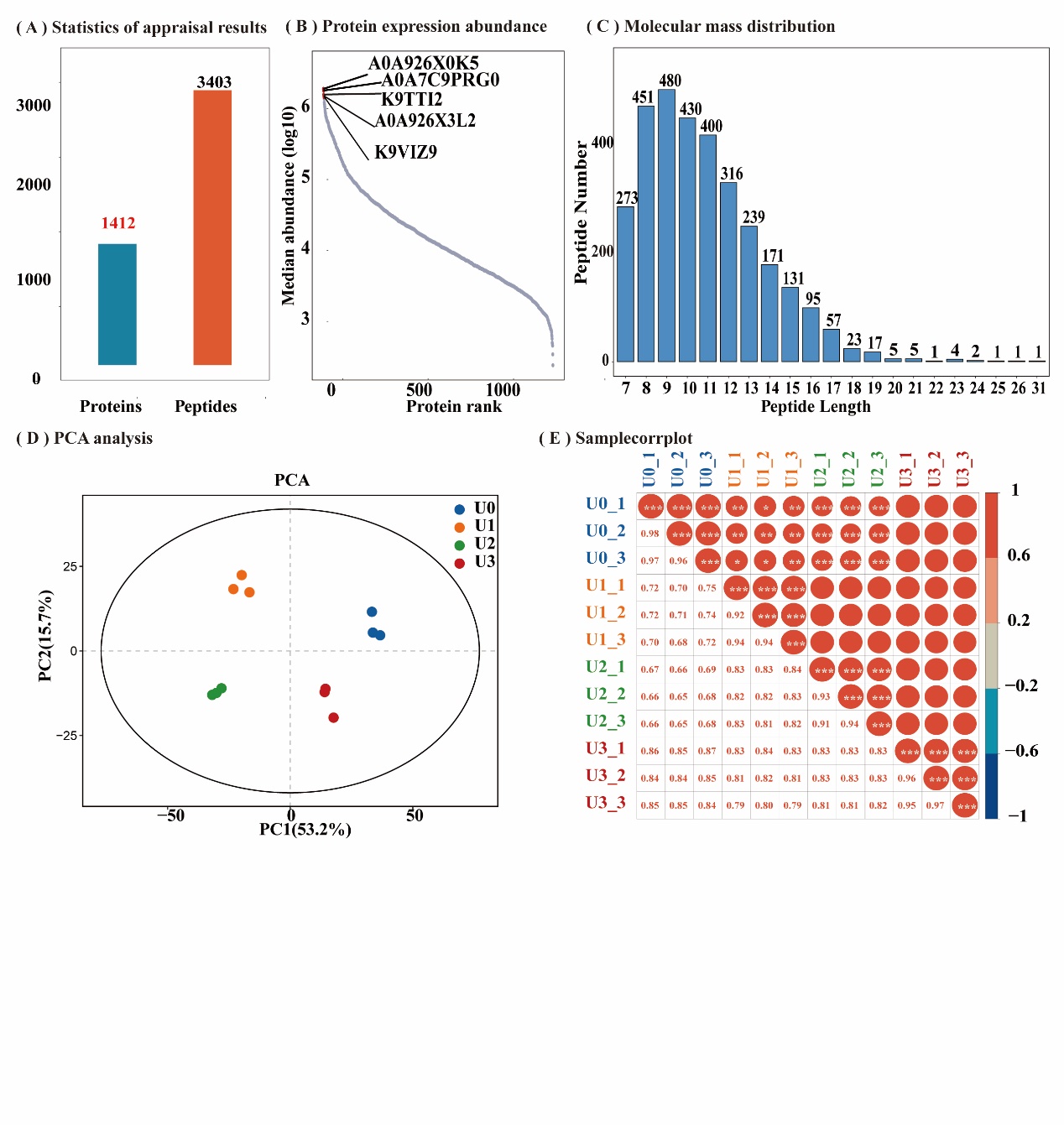


**Fig. S2.** Sequencing length distribution statistics.


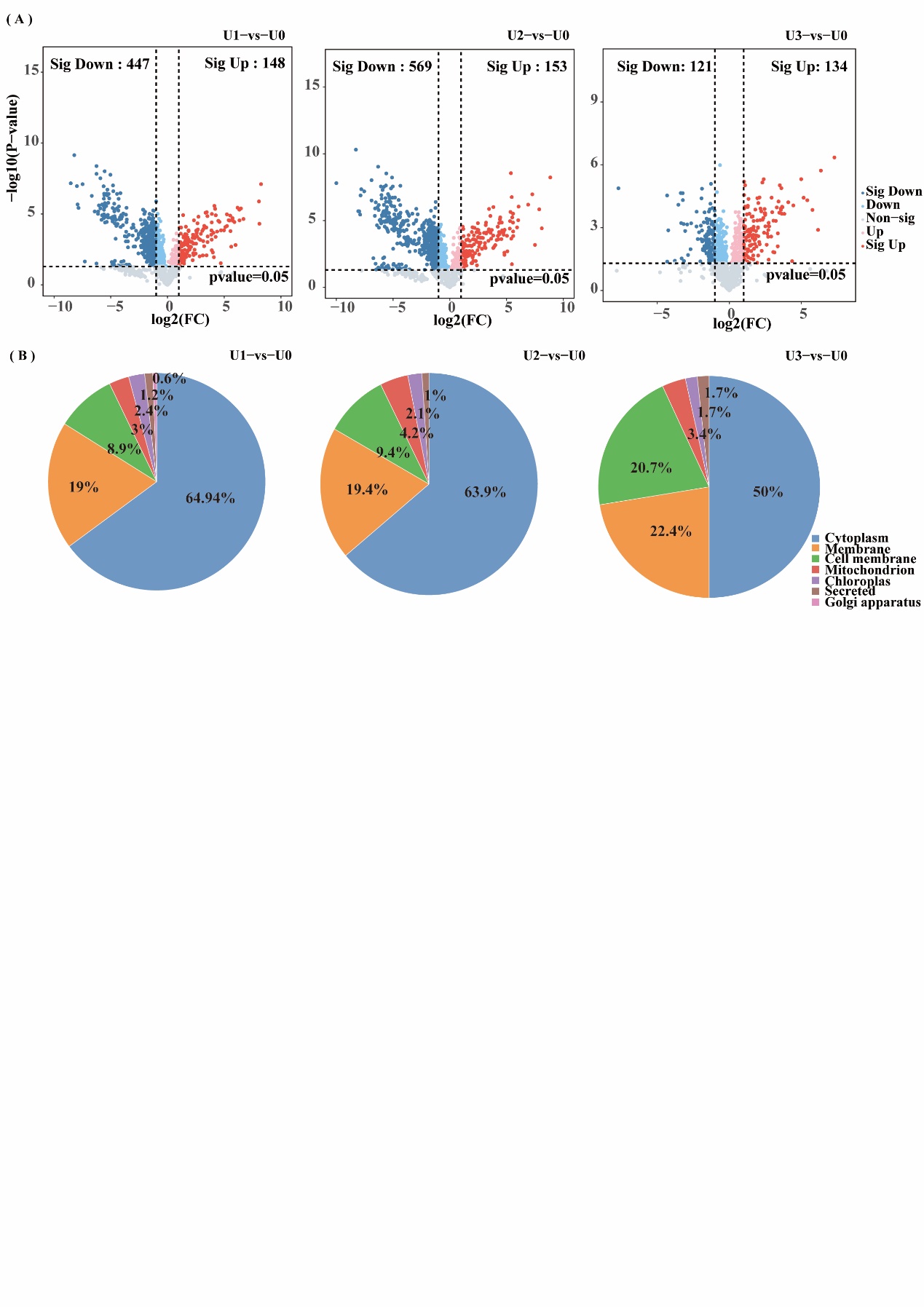


**Fig. S3.** Volcano plot analysis of differentially expressed proteins, subcellular localization.

Note：The horizonal dashed lines in the volcano plots represent p = 0.05, and the vertical dashed lines represent a fold change (FC) of 0.8 (left) and 1.25 (right). A red (blue) dot represents an upregulated (downregulated) differentially expressed metabolite.


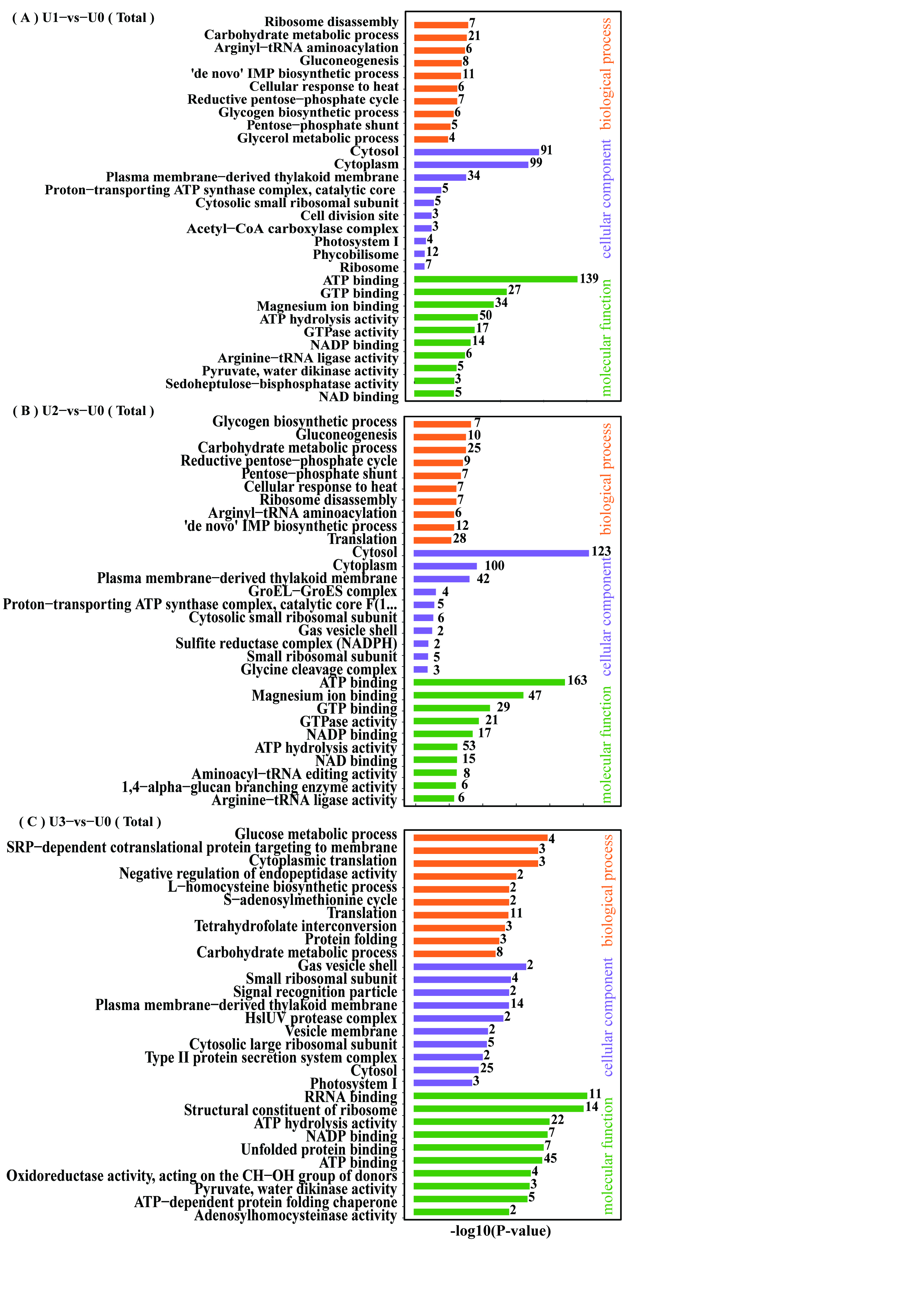


**Fig. S4.** GO Clasification


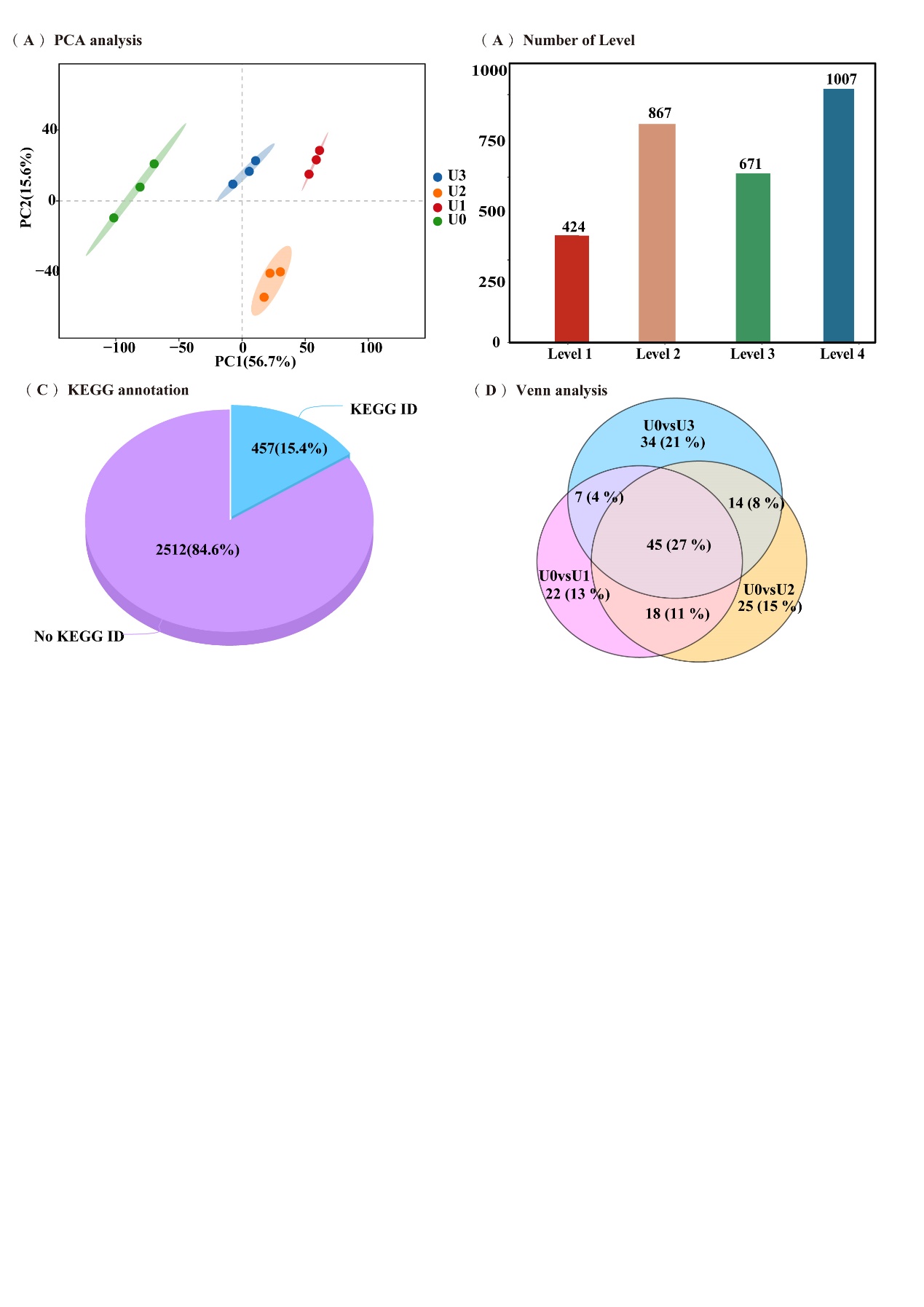


**Fig. S5.** DEMs database annotation.

## **3.6 Effect of Uranium Stress on the Metabolic Network**


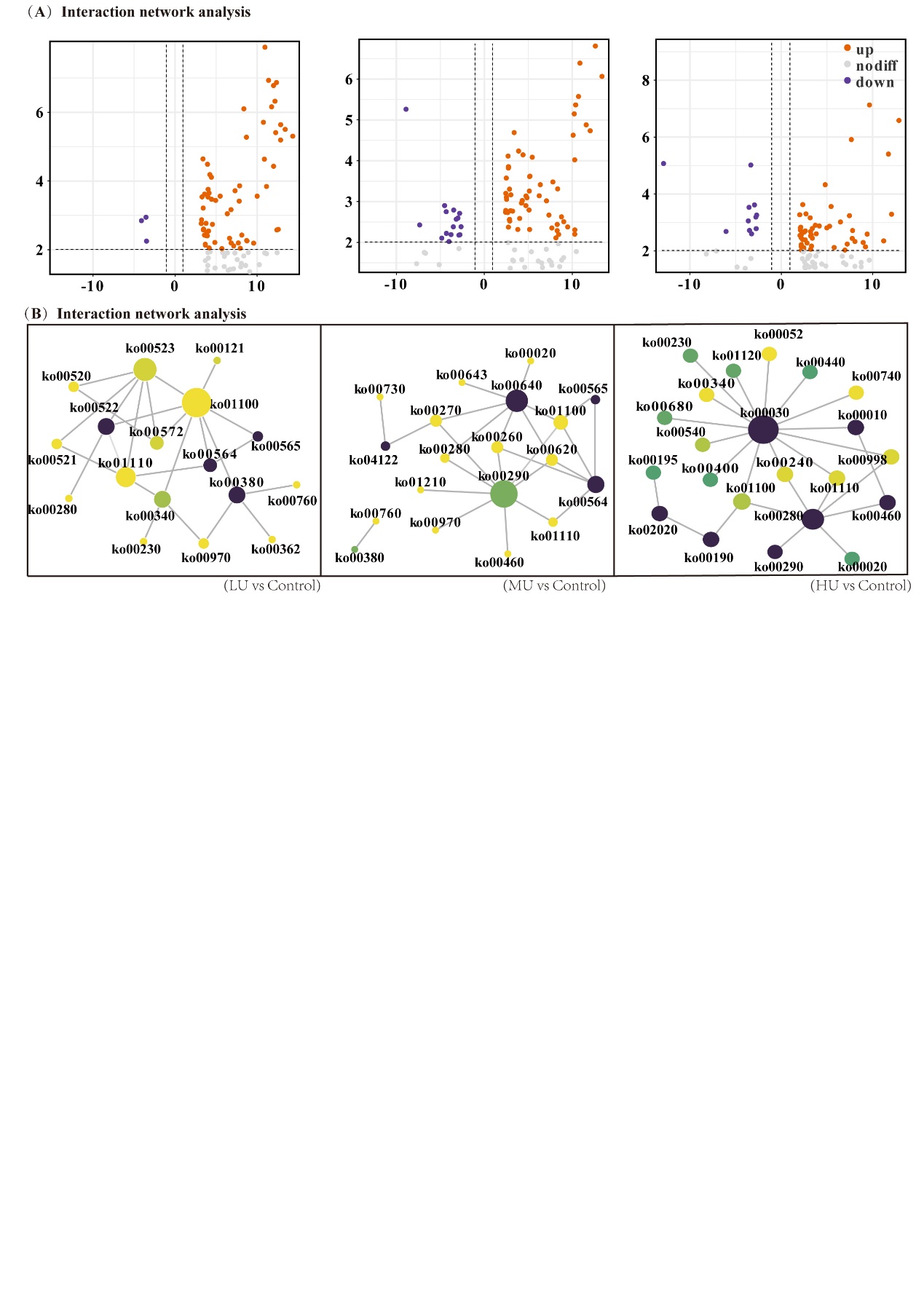


**Fig. S6.** DEMs analysis.

**Reference**

M, P.C., Miao-Hsia, L., R, H.A.J., Simone, L., 2018. Widespread bacterial protein histidine phosphorylation revealed by mass spectrometry-based proteomics. Nature methods 15, 187-190.

Kiran, K., Kumar, S.P., Yogender, A., Prakash, S.R., 2022. Secretome analysis of an environmental isolate Enterobacter sp. S-33 identifies proteins related to pathogenicity. Archives of Microbiology 204, 662-662.
